# Investigating HPV- and HPV Vaccine-Related Knowledge, Perceptions, and Information Sources among Health Care Providers in Three Big Cities in China

**DOI:** 10.3390/vaccines8030499

**Published:** 2020-09-02

**Authors:** Jie Li, Jingshi Kang, Yimeng Mao, Pinpin Zheng, Abu S Abdullah, Guoli Wu, Fan Wang

**Affiliations:** 1 School of Journalism & Communication/National Media & Experimental Teaching Center, Jinan University, Guangzhou 510632, China; lijie2007@jnu.edu.cn; 2 Journalism School, Fudan University, Shanghai 200433, China; 19110130014@fudan.edu.cn; 3 School of Public Health, Fudan University, Shanghai 200032, China; 18211020031@fudan.edu.cn (Y.M.); zpinpin@shmu.edu.cn (P.Z.); 4 Global Health Research Center, Duke Kunshan University, Kunshan 215316, China; abu.abdullah@dukekunshan.edu.cn; 5 Duke Global Health Institute, Duke University, Durham, NC 27708, USA; 6 Boston Medical Center, Boston University School of Medicine, Boston, MA 02118, USA; 7 Project HOPE, Millwood, VA 22646, USA; wuguoli@projecthope.org.cn; 8 Department of Politics, East China Normal University, Shanghai 200241, China

**Keywords:** HPV, HPV vaccine, health care provider, knowledge, perception

## Abstract

The limited access to vaccination and vaccine hesitancy are prevalent even among health care providers in less developed countries. This study assessed the relationships between (i) health care providers’ knowledge on human papillomavirus (HPV) and vaccination for HPV and (ii) their perceptions, demographic characteristics, and the use of information sources. In this large-scale online survey, participants (*n* = 1394) were recruited from hospitals of three big cities in China (Shanghai, Guangzhou, and Shenzhen). Descriptive analysis, the chi-square test, and logistic regression analysis were used to answer different research questions. Health care providers’ overall knowledge scores are consistent with their perceptions about HPV and HPV vaccine. Occupation, specialty, the type of hospitals, and the frequency of participants’ search for information using professional informational sources are the most significant characteristics that are closely associated with their knowledge of HPV and its vaccine. Targeted educational interventions are needed to improve health care providers’ engagement in the promotion of the HPV vaccine. Such interventions, besides increasing knowledge, should also emphasize the importance of appropriate information sources to acquire information.

## 1. Introduction

Human papillomavirus (HPV) is the most prevalent viral infection of the reproductive tract and is a major cause for cervical cancer [1]. Cervical cancer was the fourth most common cancer in women and was responsible for 7.5% of all female cancer deaths in 2018 [1]. Vaccines that protect against HPV are recommended by the World Health Organization (WHO) and have been approved for use in many countries. After a decade delay since U.S. FDA licensure in 2006, China approved the bivalent vaccine (2vHPV) in 2016 [2], the quadrivalent vaccine (4vHPV) in 2017, and the 9-valent vaccine (9vHPV) in 2018. The HPV vaccination is for use in females aged 9–45 years and it is not free-of-charge [3]. Up to when this survey finished in December 2018, Shanghai, Guangzhou and Shenzhen are the first group of cities in China where the HPV vaccine was available [4].

However, vaccine hesitancy, which is defined by the Strategic Advisory Group of Experts (SAGE) working group of the WHO, refers to delay in the acceptance or refusal of vaccination despite the availability of vaccination services [5]. Nowadays, this hesitation has been an increasingly common phenomenon globally and is influenced by several factors such as complacency, convenience confidence, and other reasons [6]. With respect to HPV vaccines, factors such as worries about the side effects, a low perceived efficacy of HPV vaccines and therefore no necessity of being vaccinated, and geographical accessibility and affordability could all contribute to vaccine hesitancy [7]. Evidence shows that health care providers feel ill-equipped to answer questions or uncertain to talk with those who are reluctant to be vaccinated [8], which undoubtedly causes lower vaccine uptake rates.

One effective measure to overcome hesitancy among audiences and providers is to make providers thoroughly master knowledge of HPV and HPV vaccines. This way, they can have a good understanding of the benefits and risks of being vaccinated and thus have confidence to recommend vaccination to their patients. Therefore, it is necessary to examine the knowledge, perceptions, and information sources of HPV and its vaccine among health care providers.

Being directly responsible for the recommendation and delivery of HPV vaccines to the public, the importance of health care providers has been emphasized by several studies [9,10,11,12,13]. One of the most important factors that influence the providers’ recommendation of HPV vaccination is their experience with and understanding of this vaccination. Therefore, it is worthwhile to explore the providers’ knowledge regarding HPV and HPV vaccine. There have been positive results: more than 90% of providers could correctly answer most of the questions in their surveys [14,15]. However, it seems that their knowledge regarding HPV and its vaccine does not always match with their profession. For example, a study conducted among South African doctors [16] found that the knowledge level of HPV infections was low, and they had poor awareness about HPV and HPV vaccines. Similar results were obtained with doctors in Canada [17], India [15], and China [18].

In terms of their subjective perception, some physicians tend to perceive that their knowledge is insufficient. When rating their knowledge about the risk of HPV infection and resultant cervical cancer, general practitioners (61%), obstetricians/gynecologists (28%), and pediatricians (56%) felt that their knowledge of this aspect was not sufficiently high [19]. Physicians’ knowledge and their perceptions are not always consistent and may vary from each other [18]. By examining the discrepancy between the subjective perception and objective knowledge of health care providers in three representative developed cities in China, we can lay the foundation for bridging this gap. Therefore, the first research question is the following:

RQ1: Are providers’ subjective perceptions consistent with their objective knowledge on HPV and the HPV vaccine? If not, is the variance significant?

With respect to the acquisition of relevant knowledge, most surveys on this topic assessed whether providers’ knowledge is associated with their demographics. Particularly, specialty, hospitals’ attributes, and the experience with HPV and HPV vaccination are viewed as critical variables in physicians’ knowledge. For example, there was a greater possibility that obstetricians/gynecologists knew specific oncogenic strains of HPV than the general practitioners [20], and a study by Warner and colleagues indicated that providers from institutions or universities and primary care or other have more HPV vaccination knowledge than providers from private care and hospitals, and the more patients providers saw, the more knowledge about HPV they had [21]. Furthermore, information sources could also be a significant factor that influences the physicians’ actual knowledge about HPV and HPV vaccine [22]. Therefore, research questions 2 and 3 are the following:

RQ2: Is the knowledge of providers associated with their demographic characters, especially specialty, hospital type, and practical experience? If so, which factors are the most significant?

RQ3. Is the knowledge of providers associated with the source and frequency of information acquisition? If so, which information source predicts the most significant variance?

The purpose of this study was to examine whether the knowledge of health care providers is consistent with their perceptions of HPV and HPV vaccine as well as whether the level of their knowledge is associated with their demographics, particularly their specialty, hospital attributes, practical experience, and the information acquisition frequency in three well-developed big cities of China.

## 2. Materials and Methods

### 2.1. Study Design and Implementation

We conducted this survey in Shanghai, Guangzhou, and Shenzhen, China. Shanghai is a well-known international metropolis and China’s economic and trade center. Its rapid development makes people there more aware of the advantages of HPV vaccination and more receptive to new ideas and policies, especially those related to health. Guangzhou and Shenzhen are located at Guangdong Province in Southern China. Before the HPV vaccine had been officially approved in mainland China, the benefits of the HPV vaccine had been foreseen and introduced by commercial advertisements and word of mouth in Hong Kong and Macao, which are geographically close to Shenzhen and Guangzhou. In this way, many people living in these two cities had the convenience to go to Hong Kong or Macao to get vaccinated, so the acceptance of HPV vaccination in these two cities was relatively high. Moreover, Shanghai, Guangzhou, and Shenzhen are equipped with adequate and superior medical resources compared with most other cities in China. Thus, to investigate the health care providers in these three cities will lead to a better understanding of HPV and the HPV vaccine than in most other Chinese cities.

It is very difficult to go into the Chinese hospitals to do an offline survey because of the administrative regulations and the busy work of the providers; therefore, some researchers have tried to conduct studies on Chinese medical personnel through online surveys. For instance, Zhang C and Liu Y conducted an online survey to study the salary of physicians in Chinese public tertiary hospitals, which implemented a self-administered questionnaire instrument using a smart phone platform (WeChat) [23]. Niu Chi, Guo Wei and Gu Wei also conducted an epidemiological study on the status of nutrition-support therapies by emergency physicians in China by sending online questionnaires to the emergency physicians of the participating hospitals [24]. In China, consultation regarding HPV vaccination is mainly given by gynecologists and pediatricians, while the administration of the vaccination is usually provided in community health service centers. Therefore, we use the purposive sampling in this study. First, we selected 17 maternal and child health hospitals, 31 community health centers, and 14 general and specialized hospitals in three cities. We identified a staff member as a contact person in each participating hospital. Then, we implemented a self-administered questionnaire on wjx.cn, which is the Chinese version of “Survey Monkey”. After receiving the online questionnaire link from us, the contact person in each hospital sent the link to their internal working groups of providers, including providers in General, Pediatrics, Obstetrics, and Gynecology, Preventive health care, and other specialties, inviting them to complete the questionnaire. One trained surveyor was assigned to each sample hospital to help the providers complete the questionnaire as required. To ensure that the minimum sample size was reached, we planned to get at least 15 providers from each hospital. Only those who completed this online survey were calculated, and they were in total 1394, including 427 in Shanghai, 445 in Guangzhou, and 520 in Shenzhen (missing value is 2).

### 2.2. Measures

We collected participants’ demographics by asking them about their personal information, including their city, gender, age, occupation, specialty, title, type of hospitals, and years of practical experience.

We assessed participants’ perception and knowledge on HPV and its vaccine by a set of questions. The concept of perceptions sometimes related to their perceived knowledge about some facts, such as their perceived benefits or risks about HPV vaccines [25]; however, in some cases, perceptions also were limited to their rating or confidence level of their knowledge on a certain topic [18,26]. Perception in our survey was adopted by this definition, and it was evaluated by asking three single questions about how well do they think they know about HPV/HPV vaccine/cervical cancer, which were based on previous work [19,27,28]. These questions were scored on a scale of 1–5, with 1 indicating they do not know it at all, 2 indicating that they only know it a little, 3 indicating they know it in general, 4 indicating they know it well, and 5 indicating they know it perfectly.

For the knowledge part, knowledge refers to participants’ actual knowledge level and is objective. Six questions about HPV and 4 questions about HPV vaccine were selected from the literature [27,29,30,31] and by discussion with a focus group, which was composed of experts in obstetricians and gynecologists, as well as specialists in health communication and education in China. All questions were multiple-choice questions and there was only one correct answer. For example, for the question “Which age group is at the highest risk of becoming infected with HPV?”, we offered 4 choices: A: 0–14; B: 15–24; C: 25–35; D: over 35; C was the correct answer. The instrument was scored by the total number of correct responses, and the possible scores ranged from 0 to 6 for HPV and from 0 to 4 for HPV vaccine.

During the design of the study, we wanted to assess the relationship between the knowledge level and the frequency of using different types of information sources, so we referred to available literature [22,32] and the advice given by the focus group mentioned above. Hence, we included 9 types of information sources in the questionnaire: (1) professional sources (professional organizations and scientific literature), (2) newspapers, radio, and television, (3) the Internet, (4) Chinese social media, such as Weibo and WeChat, (5) discussion with colleagues, (6) discussion with patients, (7) discussion with relatives or friends, (8) pharmaceutical companies, and (9) health administration departments. However, the distribution of data was not robust enough with very few responses in some categories. Therefore, we grouped these categories into 4 types: sources 1, 5, and 9 were merged into the item of “professional source”, sources 2, 3, and 4 were merged into the item of “media”, sources 6 and 7 were merged into the item of “interpersonal discussion”, which refers to the discussion with people who are not professionals or experts, and source 8 represents the item of “pharmaceutical companies”. Participants were asked to score these different types of information source between 1 and 5 according to their daily use habits.

### 2.3. Statistical Analysis

We used SPSS (IBM Corp., Armonk, NY, USA) 23.0 for analysis. Specifically, Pearson correlation analysis, chi-square, and logistic binary regression analysis were conducted.

Pearson correlation analysis was conducted to answer RQ1. To answer RQ2, we examined the distribution of high and low knowledge scores for HPV (classified into the “higher” and “lower” groups) based on demographic characteristics of participants by the chi-square test. To realize the daily use of information sources by providers to acquire HPV and HPV vaccine-related information, descriptive statistics analysis was used. To answer RQ3, logistic binary regression analysis was conducted. The dependent variable is binary and categorized as “lower” and “higher” knowledge level of HPV or HPV vaccine; the independent variable is the frequency of different information sources use, scores of which range from 1 to 5.

## 3. Results

### 3.1. Characteristics of the Study Population

Of the participants (*n* = 1394), 66.3% were physicians and 33.3% were nurses (missing value is 6); of the respondents, 30.6%, 31.9%, and 37.3% were from Shanghai, Guangzhou, and Shenzhen, respectively (missing value is 2). Regarding age, 18.3%, 47.0%, and 34.6% were 18–27, 28–37, and >38 years old, respectively (missing value is 2); majority were females (85.7%, missing value is 2). General practitioners, pediatricians, obstetricians, preventive care physicians, and physicians in other specialties represented 19.6%, 14.4%, 43.8%, 17.6%, and 4.5% of the study population (missing value is 2). Primary and intermediate physicians both represented more than 40% (42.0% and 46.1%, respectively), and senior physicians were a minority (11.5%) (missing value is 5). Of the participants, 45.5% had worked for 0–9 years, 32.2% had worked for 10–19 years, and only 22.2% had worked for >20 years (missing value is 2). Most participants worked in maternal and child health hospitals (39.0%) or community health centers (34.8%) (missing value is 2), and about 30% of them vaccinated over 200 patients weekly (missing value is 31).

### 3.2. Providers’ Knowledge and Perceptions about HPV and Its Vaccine

The knowledge of respondents with regard to HPV and the HPV vaccine was surveyed by asking six and four questions, respectively. The mean score on HPV was 4.47, and 54.7% of the respondents achieved a score of 5 or 6 of 6. The mean score about the HPV vaccine was 3.12, and 75% of the respondents achieved a score of 3 or 4 out of 4. When asked “How do you judge your understanding or the level of your knowledge about HPV and HPV vaccine?”, around 52.2% and 46.5% of the respondents answered that they thought they know these two topics well (score 4) and excellently (score 5). The results were shown in Table 1. Both HPV (*p* < 0.01) and HPV vaccine knowledge scores were significantly correlated with the respondents’ perception of these aspects, and the overall knowledge score was similarly associated with the general perception (*p* < 0.01).

### 3.3. The Relationship between Providers’ Knowledge and Demographics

As shown in Table 2, providers from Shenzhen achieved significantly higher scores on the HPV knowledge questions than providers from Guangzhou. The accuracy of pediatricians was the lowest, while obstetricians and gynecologists answered the most questions correctly. In addition, physicians normally had a better understanding about medical topics than nurses, and senior physicians knew more than intermediate and primary physicians.

As shown in Table 3, only 56% of the pediatricians obtained higher scores for HPV vaccine knowledge. However, over 90% of those who work in preventive health care reached a higher score. As for the types of hospital, the lowest percentage of participants in general and specialist hospitals scored higher scores for HPV vaccine knowledge but over 85.2% of providers in the community health service center had a better grasp about this topic. Besides, providers of middle age and those with an intermediate title had higher knowledge scores about the HPV vaccine than the older providers and seniors. Predictably, higher knowledge scores on the HPV vaccine were obtained by physicians than by nurses, by providers who vaccinate over 200 patients weekly than by those who vaccinate < 200 patients, and by providers aged 28–37 than by the rest.

### 3.4. Relationship between Providers’ Knowledge and Their Information Sources

The mean values (score range is from 1 to 5) of the frequencies of different kinds of information sources used are the following: 3.47 for professional sources (including professional readings and discussions with colleagues), 3.21 for media (including mass media, websites, and social media such as WeChat and Weibo), 3.04 for interpersonal discussion (including discussions with friends working in other fields and patients), and 2.70 for pharmaceutical companies. Clearly, professional and media sources are the most frequently used information sources for this topic.

Table 4 shows that knowledge scores on HPV and the HPV vaccine were significantly positively associated with different frequencies of professional information sources use. It means that higher frequencies of professional sources use were consistent with reaching a higher knowledge level on the two aspects. However, with respect to other information sources, no significant differences were observed between the frequency of use and the knowledge level for HPV and its vaccine.

## 4. Discussion

In this study, we analyzed the knowledge, perception of HPV and HPV vaccine, and the use of information sources for relevant resources from the health care providers’ perspective by conducting a large-scale survey with 1394 participants covering three big cities in China. First, we examined whether providers’ knowledge level were consistent with their perceptions about HPV and HPV vaccine; then, the variance of providers’ knowledge level based on their demographic characteristics such as city, specialty, and hospital type were analyzed according to 6 questions for HPV and 4 questions for HPV vaccine; finally, the relationship of providers’ knowledge level regarding HPV and its vaccine and their frequency of different information source use was verified.

Nowadays, it has been emphasized repeatedly that receiving a recommendation from a health care provider is one of the most significant and consistent factors that influence the audience’s decision on HPV vaccination [21,33,34]. However, on the one hand, vaccine hesitancy is pervasive in ordinary people’s views. Concerns such as vaccine safety and the necessity of vaccination are the most reported reasons for vaccine refusal [35]. Therefore, it is critical for people to determine the benefits of being vaccinated when they receive the recommendation of their health care providers. On the other hand, hesitancy is also widespread in providers’ attitudes and their clinical practice. Moreover, barriers such as embarrassment to discuss sexual health topics with adolescents’ parents and worries about initiating time-consuming or confrontational debates among providers may impede providers’ willingness of recommendation [11,36]. Hence, probably only a comprehensive and in-depth understanding of the relevant knowledge can strengthen the motivation of recommendation. Therefore, improving providers’ knowledge on HPV and HPV vaccine could be regarded as a crucial and practical way to reduce both providers and patients’ uncertainties and enhance their belief in vaccination, and this paper is a preliminary attempt for exploring this issue.

Generally, in this study, respondents had good knowledge of HPV and its vaccine, which fairly coincided with their own perceptions about their understanding of these topics.

It is worth noting that far more providers from Shenzhen than from Shanghai and Guangzhou achieved higher HPV knowledge scores. However, it is evident that the medical resources and the level of medical treatment of the latter two cities are superior to those of Shenzhen according to China’s hospital rankings, which are professional and acknowledged widely in China [37]. Therefore, it seems that the health care providers’ HPV-related knowledge level is not always proportional to the general medical level of their cities. We speculate that the better HPV-related knowledge among health care providers in Shenzhen may due to three reasons. Firstly, it is likely attributable to the geographical location of Shenzhen, as it is located the closest to Hong Kong, where the HPV vaccine was introduced in 2004 [38] and has been covered by mass media since then; thus, it is reasonable that citizens of Shenzhen acquired relevant information by means of interpersonal communication and some public service advertisements from Hong Kong [39]. Secondly, it is possibly associated with the medical system. In Shenzhen, the implementation plan for the comprehensive reform of public hospitals was published by the Health and Family Planning Commission on 8 June 2015, making Shenzhen the first pilot city in China to print and distribute the comprehensive reform plan of public hospitals, and since then, a series of community hospitals equipped with sufficient medical staff have been established, which offered valid support for citizens getting vaccinated in adjacent areas. Thirdly, and perhaps the most important factor, Shenzhen has included HPV vaccination in citizens’ medical insurance since 10 April 2018. Therefore, the increasing number of people who get vaccinated could promote the providers’ willingness to learn about HPV and HPV vaccination. Based on these reasons, hospitals and government subdivisions of other cities should consider including HPV vaccination in the medical system to ensure that HPV-related knowledge can be disseminated through organizational communication, so that (i) more comprehensive news on HPV and HPV vaccination will be presented by media and (ii) HPV vaccination will be considered more legitimate by government agencies and more rational by citizens.

With respect to different specialties where respondents worked, it is not surprising that most obstetricians and gynecologists and respondents who worked in preventive health care departments obtained better knowledge scores for HPV and its vaccine, respectively. However, most of the pediatricians obtained lower knowledge scores for both HPV and its vaccine, which would hinder their recommendation of vaccination to the parents of adolescents or teenagers who are the target group for vaccination promotion. Previous studies indicated that the pediatricians’ recommendation is a key factor that strongly influences the vaccination decisions of adolescents and parents for their children at the right age [40]. However, on one hand, most media coverage, including mass media and social media, aims vaccination information at adult women, such as female college students or female working people, ignoring the younger population to a large extent. On the other hand, it is hard for parents to talk about HPV-related topics with their children. HPV is associated with sexual organs, while in China, parents’ consciousness of sex education is weak [41,42] and many parents feel embarrassed to talk about it with their children. This is an issue that deserves attention that the adolescents’ knowledge of HPV and its vaccine might be inaduquate; therefore, studies that investigating the possibilities to improve HPV-related knowledge among pediatricians and to increase the vaccination rate among adolescents are needed.

In terms of the type of hospital, it seems that participants who worked at general and specialist hospitals had the best understanding of HPV (the result was not significant and was therefore not shown in Table 2), while our results also indicated that they had the lowest understanding of the HPV vaccine. This reverse knowledge gap requires further investigation. Strikingly, even though the providers who worked at community health service centers are the least on getting higher knowledge scores about HPV among three types (not significant), they achieved significantly higher knowledge scores for the HPV vaccine than other groups, which might be attributable to the HPV vaccination policies in China. Our findings imply that there may exist a potential serious lack of knowledge of the “beneficial effects” and “risks”, which means that providers in general hospitals normally have good knowledge about the risk of not vaccinating but insufficient knowledge about the benefits of vaccination. Conversely, health care providers in community hospitals generally had good vaccination-related knowledge, yet they did not understand HPV pathology well. When patients visit general hospitals, they can acquire more clinical knowledge about HPV, but there is a shortage of vaccination-related knowledge, and when they are in a community hospital setting, vaccination-related suggestions are given sufficiently, but the available information about HPV may be absent. Both situations would affect the patients’ willingness and decisions to get vaccinated and would also cause vaccine hesitancy. Accordingly, communication strategies and the distribution of information should be improved. In terms of policies, the range of officially recommended hospitals for vaccination should be expanded, so that vaccinations can also be given at general and specialist hospitals. As regards the distribution of information, it will be beneficial to strengthen the supply of information about HPV vaccination to health care providers in higher-level hospitals to increase the accessibility of vaccination information. In turn, training on the clinical pathology of HPV should be offered to health care providers in community hospitals to improve the capacity of primary hospitals.

Furthermore, regarding the age and title, senior providers generally obtained higher scores on the knowledge of HPV, while intermediate physicians and middle-aged (28–37) respondents obtained higher scores on the knowledge of HPV vaccine. This difference may be caused by the fact that the participants who take charge of vaccination are normally younger. In this case, regular training about HPV should be offered to young and intermediate health care providers, which is a key step for reminding them of their deficiency on the knowledge of HPV pathology and inspiring them to take the initiative in this learning process. By this way, they can not only offer suggestions about vaccines to patients, but also can provide advice on the dangers of HPV. The resulting fears could probably be useful to decrease the potential vaccine hesitancy of the patients.

Health care providers, as professionals, are the transmitters of medical information to the audience. Therefore, their knowledge and perceptions are important factors in influencing vaccine acceptability [43]. Many studies emphasized the need for more education for providers on HPV-related topics [14,25,27]. In this paper, we noted that interventions should not only aim at raising providers’ knowledge level on HPV and its vaccine, but also should be based on providers’ different characteristics such as specialty and the type of their hospitals, which would benefit them more by equipping them with confidence and adequate knowledge on recommendation of vaccination. Moreover, such interventions can effectively relieve unnecessary concerns in the general public.

Furthermore, the use of more professional information sources was associated with more accurate HPV-related knowledge. Frequent discussions with peers on relevant issues could also be useful. Other information sources, such as media, may be crucial in information seeking, but could not facilitate a higher level of knowledge acquisition. Based on these results, we recommend that the physicians and nurses should search for knowledge from professional journals and other reliable professional sources.

There were some limitations to this study. Due to the nature of the survey link distribution, we did not know the exact number of providers who has received the survey link or in fact browsed the link that we have provided. In addition, those who participated in the survey might be those who were interested in the topic and were more aware of the HPV situation in China and elsewhere. Thus, the results have limited generalizability and should be interpreted carefully. Another limitation of this study is that all the participants in our sample pool were health care providers from developed cities in China. More health care providers might be short of relevant knowledge in underdeveloped cities; we plan to investigate this population in our future studies. Additionally, all data collected in this study were self-reported, and that may generate bias.

## 5. Conclusions

In this large-scale survey, we examined the health care providers’ knowledge and perceptions on HPV and the HPV vaccine, as well as whether their self-reported perceptions of their own knowledge were consistent with their knowledge in the context of vaccine hesitancy. We also analyzed whether their knowledge levels were associated with demographics, such as specialty, hospital attributes, practical experience, and the frequency of information acquisition from different sources. We found that participants’ perceptions of their knowledge of HPV and HPV vaccine were generally consistent with their knowledge scores. In addition, city, specialty, title, and type of hospital are significantly associated with the knowledge levels of health care providers either on HPV or HPV vaccine, or both, which reflects some phenomenon or shortage of current situations of HPV and HPV vaccination in China. The providers should use professional sources more often to search for knowledge on the two aspects.

## Figures and Tables

**Table 1:** Pearson correlation between the knowledge score and the self-reported perception of respondents’ knowledge on human papillomavirus (HPV) and its vaccine.

Variables	Knowledge Score on HPV	Knowledge Score on HPV Vaccine	Total Knowledge Score
Perception score of HPV	0.254 **		
Perception score of HPV vaccine		0.238 **	
Total perception score			0.319 **

** meaning the *p* value is significant at 0.01 level.

**Table 2:** Distribution of respondents between “lower” and “higher” groups based on their HPV knowledge scores.

	Knowledge Score on HPV ^e^			
Demographics	Lower (45.3%)	Higher (54.7%)	χ^2^	*p* Value
City ^a^			29.450	0.000 ^‡^
Shanghai	178 (41.9%)	247 (58.1%)		
Guangzhou	247 (55.5%)	198 (44.5%)		
Shenzhen	202 (38.8%)	318 (61.2%)		
Occupation ^b^			41.405	0.000 ^‡^
Physician	359 (38.9%)	564 (61.1%)		
Nurse	265 (57.1%)	199 (42.9%)		
Specialty ^c^			57.297	0.000 ^‡^
General	133 (48.7%)	140 (51.3%)		
Pediatrics	131 (65.2%)	70 (34.8%)		
Obstetrics and gynecology	220 (36.1%)	389 (63.9%)		
Preventive health care	108 (44.1%)	137 (55.9%)		
Other	35 (56.5)	27 (43.5%)		
Title ^d^			13.004	0.002 ^†^
primary	289 (49.4%)	296 (50.6%)		
Intermediate	283 (44.0%)	360 (56.0%)		
Senior	54 (33.8%)	106 (66.3%)		

Only significant values are presented. ^†^: *p* value is significant at the level of 0.01. ^‡^: *p* value is significant at 0.001. The number of participants in every demographic characteristic is less than the total number of participants we surveyed because of the missing values. Specifically, the total number of participants we surveyed is 1394; however, the number of participants that accurately filled in items “^a^”, “^b^”, “^c^”, “^d^”, and “^e^” in Table 2 effectively is 1392, 1388, 1392, 1389, and 1390 respectively (the corresponding missing values are 2, 6, 2, 5, and 4). Therefore, the total number of every demographic character represented in this table is influenced both by its missing values and the missing values of HPV. The preventive health care providers in ^c^ refers to the providers who professionally work in prevention and health care department; ^d^ was categorized as three options based on the title system of Chinese professional health providers.

**Table 3:** Distribution of respondents between “lower” and “higher” knowledge scores based on the HPV vaccine knowledge score.

Demographics	Knowledge Score on HPV Vaccine ^g^			
Demographics	Lower (24.6%)	Higher (75.4%)	χ^2^	*p* Value
Occupation ^a^			10.397	0.001 ^†^
Physician	203 (22.0%)	718 (78.0%)		
Nurse	139 (30.0%)	325 (70.0%)		
Age (years) ^b^			7.328	0.026 *
18–27	79 (31.1%)	175 (68.9%)		
28–37	147 (22.5%)	506 (77.5%)		
>38	117 (24.3%)	365 (75.7%)		
Specialty ^c^			77.083	0.000 ^‡^
General	52 (19.0%)	221 (81.0%)		
Pediatrics	88 (44.0%)	112 (56.0%)		
Obstetrics and gynecology	158 (26.0%)	450 (74.0%)		
Preventive health care	24 (9.8%)	221 (90.2%)		
Other	21 (33.3%)	42 (66.7%)		
Title ^d^			10.166	0.006 ^†^
Primary	168 (28.8%)	416 (71.2%)		
Intermediate	134 (20.9%)	507 (79.1%)		
Senior	40 (24.8%)	121 (75.2%)		
Types of hospital ^e^			42.135	0.000
Community health service center	72 (14.8%)	413 (85.2%)		
General and specialist hospitals	120 (33.1%)	242(66.9%)		
Maternal and child health care institution	151 (27.9%)	391 (72.1%)		
Average number of vaccinations weekly ^f^			23.651	0.000
<200	268 (28.2%)	682 (71.8%)		
≥200	65 (15.9%)	345 (84.1%)		

Only significant values are presented. *: *p* value is significant at the level of 0.05. ^†^: *p* value is significant at the level of 0.01. ^‡^: *p* value is significant at the level of 0.001. The number of participants in every demographic characteristic is less than the total number of participants we surveyed because of the missing values. Specifically, the total number of participants we surveyed is 1394; however, the number of participants that accurately filled in items “^a^”, “^b^”, “^c^”, “^d^”, “^e^”, “^f^”, and “^g^” in Table 3 is 1388, 1392, 1392, 1389, 1392, 1363, and 1389, respectively (the corresponding missing values are 6, 2, 2, 5, 2, 31, and 5). Therefore, the total number of every demographic character represented in this table is influenced both by its missing values and the missing values of HPV vaccines.

**Table 4:** The relationship between the knowledge level and frequency of different information sources use.

Information Sources	Variables in the Equation (HPV)			
	**B**	**Wald**	***p*** **Value**	**Exp (B)**
Professional sources	0.664	52.410	0.000 ^‡^	1.942
Media	−0.106	1.834	0.176	0.899
Discussion	−0.083	0.942	0.332	0.921
Pharmaceutical companies	−0.079	1.659	0.198	0.924
Constant	−1.295	29.518	0.000	0.274
	**Variables in the equation (HPV Vaccine)**			
	B	wald	*p* value	Exp (B)
Professional sources	0.478	22.352	0.000 ^‡^	1.614
Media	0.120	1.791	0.181	1.127
Discussion	−0.064	0.421	0.517	0.938
Pharmaceutical companies	0.016	0.050	0.824	1.0016
Constant	−0.726	7.777	0.005	0.484

^‡^: *p* value is significant at the level of 0.001.
